# Bi‐ and Mono‐Allelic RFC1 Expansion in a North American Cohort With Idiopathic Axonal Neuropathy

**DOI:** 10.1002/acn3.70462

**Published:** 2026-07-05

**Authors:** Amro M. Stino, Lavanya Muthukumar, Evan L. Reynolds, Peter Todd, Sinem Ovunc, Sheng Chih Jin, Zitian Tang, Simone Thomas, PNRR Study Group, Sarah Berth, Vinay Chaudhry, David Cornblath, Leana Doherty, Lindsey Hayes, Hristelina Ilieva, Thomas Lloyd, Mohammad Khoshnoodi, Brett McCray, Brett Morrison, Bipasha Mukherjee‐Clavin, Lyle Ostrow, Michael Polydefkis, Ricardo Roda, Charlotte Sumner, Ahmet Höke, Brian C. Callaghan

**Affiliations:** ^1^ Department of Neurology University of Michigan School of Medicine Ann Arbor Michigan USA; ^2^ Department of Epidemiology and Biostatistics Michigan State University East Lansing Michigan USA; ^3^ Veterans Administration Ann Arbor VA Healthcare Ann Arbor Michigan USA; ^4^ Department of Genetics Washington University in St. Louis School of Medicine St. Louis Missouri USA; ^5^ Department of Neurology Johns Hopkins University School of Medicine Baltimore Maryland USA

**Keywords:** CANVAS, electrodiagnostic, phenotype, polyneuropathy, RFC1

## Abstract

**Objective:**

RFC1 biallelic repeat expansion is increasingly recognized as a cause of chronic idiopathic axonal polyneuropathy (CIAP), but it remains challenging to know who to test. This study aims to determine the prevalence of biallelic and monoallelic RFC1 expansions and their corresponding neuropathy phenotypes in CIAP patients and identify discriminators of RFC1 expansion status.

**Methods:**

The cross‐sectional Peripheral Neuropathy Research Registry was queried for individuals with CIAP and tested for RFC1 expansions. Associations between RFC1 biallelic and monoallelic status and clinical history, neurologic examination findings, fiber type involvement (exam‐based), electrodiagnostic (EDX) patterns (sensory, sensorimotor, motor), and the presence of sensory neuronopathy (Camdessanche criteria) were determined.

**Results:**

A total of 788 participants were enrolled in the study, of whom 18 had biallelic RFC1 expansions (2.3%) and 62 had monoallelic RFC1 expansions (7.9%). All biallelic RFC1 patients had a mixed fiber neuropathy, 27.8% had weakness on examination, and 44.4% had motor EDX abnormalities. Our study identified five factors associated with RFC1 biallelic status: (1) abnormal upper limb vibration (OR 4.65, 95% CI 1.20–18.13), (2) abnormal sensory EDX studies (OR 4.65, 95% CI 1.11–24.29), (3) sensory‐only polyneuropathy on EDX (OR 7.17, 95% CI 1.69–38.15), (4) sensory neuronopathy (OR 3.64, 95% CI 1.16–11.19) (AUCs = 0.80–0.81 for biallelic and 0.69–0.71 for monoallelic), and (5) body mass index (OR 0.88, 95% CI 0.78–0.97).

**Interpretation:**

The prevalence of biallelic RFC1 expansion is lower in this North American CIAP population compared to previous studies. Motor involvement was frequent. Discrimination of biallelic status was good, but poor for monoallelic status.

## 1 Introduction

Chronic idiopathic axonal polyneuropathy (CIAP) is a slowly progressive axonal neuropathy that constitutes roughly one‐third of all cases of polyneuropathy [1]. In the last 5 years, biallelic expansion of AAGGG pentanucleotides (TTCCC in the transcription sense) in the second intron of the replication factor complex subunit 1 (RFC1) has emerged as a recognized cause of sensory neuropathy or neuronopathy based on sentinel work published by Curro et al. in 2021 on behalf of a joint Italian and British consortium [2]. RFC1 AAGGG expansion associated sensory neuropathy (henceforth referred to as RFC1 expansion associated neuropathy) is best understood within the broader context of disorders associated with Cerebellar Ataxia Neuropathy and Vestibular Areflexia Syndrome (CANVAS). The full CANVAS syndrome is the tip of the iceberg of RFC1 associated disorders, thought to constitute only 28% of total RFC1 associated cases, with isolated sensory neuropathy forming the base of the iceberg, constituting roughly 42% of RFC1 associated cases [2].

Much interest has focused on the phenotypic characterization of RFC1 biallelic expansion associated sensory polyneuropathy, including its potential overlap/misdiagnosis with autoimmune neuropathy [3] as well as its role in isolated sporadic pure cerebellar ataxia [4] and multisystem atrophy [5]. Starting with the 2021 Curro paper and further supported by work from Tagliapietra et al. in Italy in the same year [6], it became clear that RFC1 biallelic sensory neuropathy frequently manifests as a sensory‐only length‐dependent neuropathy, despite pathologic and phenotypic data to suggest that it is truly more of a sensory neuronopathy [7]. Specifically, these studies found 34%–53% of all idiopathic sensory neuropathy patients in their respective cohorts had biallelic expansions in RFC1, but that only 18% of sensory predominant and 0%–2% of sensorimotor neuropathy patients had expansions. Studies from New Zealand demonstrated a lower prevalence ranging from 10% to 11% but identified small ultrasonographic nerve cross‐sectional area as a predictor of RFC1 biallelic expression in CIAP [8, 9], especially in the setting of chronic cough and/or a sensory neuronopathy pattern on EDX [8]. The predictive value of reduced cross‐sectional area on ultrasonography in discerning RFC1 biallelic expansion associated neuropathy from idiopathic axonal neuropathies [10, 11] and even Charcot–Marie–Tooth type 2 [12] has been shown in other studies as well.

Identifying CIAP patients likely to harbor RFC1 repeat expansion is of potential value as it would allow for a more targeted, phenotype‐driven, and cost‐effective approach to testing, since RFC1 genetic testing is costly and not included in most commercial panels. While both 2021 papers focused on the clinical and EDX phenotyping of biallelic RFC1 repeat expansions in European cohorts with chronic idiopathic axonal polyneuropathy (CIAP), no studies have assessed biallelic RFC1 prevalence or phenotype in North America. In addition, no studies to date have specifically described the clinical and EDX phenotype of those with monoallelic RFC1 expansions (or RFC1 carrier status), despite an increased prevalence of RFC1 carrier status in CIAP [13].

Therefore, the objective of this study was to determine the prevalence of biallelic and monoallelic RFC1 expansions and their corresponding neuropathy phenotypes in a North American cohort of participants with well‐phenotyped CIAP. We also assessed the clinical and EDX factors associated with biallelic and monoallelic RFC1 expansion status to aid clinicians in determining which patients should undergo genetic testing.

## 2 Methods

### 2.1 Population and Study Design

The cross‐sectional Peripheral Neuropathy Research Registry (PNRR) is a nationwide registry in the United States spanning 7 academic medical centers consisting of expertly‐phenotyped participants with diabetic neuropathy or CIAP. For the purposes of our present study, we only selected participants with CIAP who were recruited at Johns Hopkins Hospital between 2011 and 2023. Participants with diabetes were included if the examining neuropathy expert did not believe diabetes to be contributory to the patient's neuropathy, such as neuropathy onset before diabetes, thus rendering the diagnosis most likely CIAP. Also included in our CIAP cohort were participants with prediabetes, obesity, and metabolic syndrome. As part of the expert assessment and phenotyping of eligible participants, participants complete a neuropathy symptom questionnaire, which also includes medical, family, and social history. The neurologic examination comprises testing of proprioception (normal, reduced, absent), vibration (defined as normal, reduced, or absent, using the Rydel Seiffer technique and age‐adjusted norms), and pinprick sensation (normal, reduced, absent) at the toes, ankles, and fingers. Motor strength testing (Medical Research Council), deep tendon reflex assessment, balance (Romberg), and gait examination (including tandem) are also performed. Every participant had one glycemic test (hemoglobin A1c, fasting glucose, or 2‐h oral glucose tolerance test), B12 level, and serum protein electrophoresis with immunofixation. More extensive neuropathy lab testing, such as for autoimmune etiologies, was not required but was left to the discretion of the evaluating investigator. Large nerve fiber biopsy was not required nor routinely performed. Ultimately, a diagnosis of CIAP was predicated upon the expert clinical opinion of the evaluating investigator. Nerve conduction studies (NCS) and skin biopsy evaluation of the distal leg site to evaluate intraepidermal fiber density (IENFD) are performed in some but not all patients. Of note, a previously published study from our group demonstrated high similarity between participants with or without confirmatory NCS or IENFD testing in our cohort [14].

### 2.2 Outcome

#### 2.2.1 RFC 1 Testing

RFC1 expansions were assessed in all 788 participants using both whole‐genome sequencing (WGS; Illumina, 30× coverage) and repeat‐primed PCR (RP‐PCR). Genomic DNA was extracted from whole blood, buffy coat, or saliva samples. For initial detection, we applied our previously described bioinformatics pipeline, which utilizes a highly accurate hybrid approach combining ExpansionHunter Denovo and ExpansionHunter [15]. We then classify genotypes through unsupervised clustering.

To further validate and confirm carrier status and genotypes, we conducted PCR assays as previously described [2, 16, 17]. Briefly, we performed RP‐PCR targeting AAGGG expansion to identify carriers, with samples showing a smear or distinct band on gel electrophoresis undergoing capillary electrophoresis (Genewiz Inc.), analyzed using Peak Scanner Fragment Analysis Software to confirm expansions. Samples positive for AAGGG expansion were subsequently tested for wild‐type allele status using AAAAG in RP‐PCR. Those showing bands in the AAAAG RP‐PCR were classified as monoallelic carriers, whereas samples without bands were classified as biallelic carriers.

### 2.3 Potential Phenotypic Predictors

Clinical self‐reported, clinical exam, and EDX data points were collected for enrolled participants as outlined below.

#### 2.3.1 Clinical Self‐Report

Participants reported pain intensity related to CIAP on a scale of 0–10 (0 = no pain, 10 = most pain) using a 10‐point numeric rating scale (NRS). In addition, other symptoms of interest included the subjective presence or absence of allodynia, numbness, paresthesia, contractions, gait imbalance, and subjective weakness.

#### 2.3.2 Clinical Examination

Regular gait, tandem gait, and Romberg testing were reported as normal or abnormal. Pinprick, vibration, and proprioception data were collected for both great toes, ankles, and DIP joints. Deep tendon reflexes were deemed normal or abnormal (reduced/absent) for the Achilles, patellae, brachioradialis, biceps, and triceps. Strength testing was deemed normal or abnormal (irrespective of degree of weakness) for the great toe extensors (extensor hallucis longus), ankle dorsiflexors, and finger abductors. If participants had any abnormal finding on these examinations (pinprick, vibration, proprioception, reflexes, and strength), we classified them as abnormal for that test. We also determined whether any upper extremity abnormalities were present for these examinations and whether participants had abnormalities in all reflexes tested. Of note, clinical examination and phenotyping were completed at the time of the blood draw, which was subsequently used for genetic testing.

#### 2.3.3 Neuropathy Severity

The Total Neuropathy Score‐reduced (TNSr), which consists of 5 components (symptom extension, pin sensibility, vibration sensibility, muscle strength, and tendon reflexes), ranges from 0 (normal) to 20 (severe neuropathy). Mild (1–8), moderate (9–15), or severe (16–20) neuropathy categorizations were made [18].

#### 2.3.4 Fiber Type Classification

The Analgesic, Anesthetic, and Addiction Clinical Trial Translations, Innovations, Opportunities and Networks (ACTTION) diagnostic criteria were used to classify fiber type involvement based on neurological signs [19]. Small fiber neuropathy was defined as the presence of decreased pinprick sensation with normal vibration and proprioception. Large fiber neuropathy was defined as decreased vibration or proprioception with normal pinprick. Mixed fiber neuropathy was defined as decreased pinprick sensation and decreased vibration or proprioception. The PNRR protocol outlines pinprick, vibration, and proprioception examination techniques and data collection methods [20].

#### 2.3.5 Electrodiagnostic Data

Sensory nerve action potential and conduction velocity data were collected for sural, ulnar, median, and radial sensory nerves. Compound muscle action potential and conduction velocity data were similarly collected for peroneal and ulnar motor nerves. We determined whether participants had any sensory nerve conduction study abnormality or if they had any motor nerve conduction study abnormality.

#### 2.3.6 Polyneuropathy Pattern

Using the above collected EDX data, patients were stratified into one of four possible patterns: (1) sensory polyneuropathy, (2) sensorimotor polyneuropathy, (3) motor polyneuropathy, or (4) normal. Sensory was defined as abnormal sural velocity or amplitude in the setting of normal peroneal motor parameters. Sensorimotor was defined as abnormal sural sensory parameters (velocity or amplitude) and abnormal peroneal motor parameters (velocity or amplitude). Motor was defined as abnormal peroneal motor velocity or amplitude in the setting of normal sural sensory parameters (note that patients with co‐existing lumbosacral radiculopathy were excluded from the PNRR).

#### 2.3.7 Sensory Neuronopathy Status

Sensory neuronopathy was defined based on the Camdessanche criteria, requiring a score of > 6.5 points for the establishment of a “possible” sensory neuronopathy [21]. Minor modifications were implemented for our study purposes given constraints inherent in the registry design. For criterion A, namely the “presence of ataxia in the lower or upper limbs at onset or full development of neuropathy,” we fulfilled this if the patient had abnormal Romberg testing or tandem gait (finger‐to‐nose and heel‐to‐shin were not collected). For Criterion B, namely “the asymmetrical distribution of sensory loss at onset or full development,” asymmetry in pinprick, vibration, or position sense at the great toes or distal interphalangeal joints qualified. For criterion C, “sensory loss not restricted to lower limbs,” abnormal finger vibration, position, or pinprick sense on exam sufficed. For criterion D, the absence of median, ulnar, or radial sensory EDX responses, or reductions in EDX amplitude (< 30% lower limit of normal) in the three above‐mentioned nerves fulfilled this criterion. Of note, criterion D was also used in isolation to separately define non‐length dependent EDX sensory loss. Finally, for criterion E, “less than two nerves with abnormal motor nerve conduction studies in the lower limbs,” a normal peroneal motor nerve sufficed as the tibial motor nerve was not routinely tested.

### 2.4 Statistical Analysis

Descriptive statistics were used to summarize the demographic information, metabolic risk factors, and clinical and EDX phenotyping characteristics stratified by RFC1 status (monoallelic vs. biallelic vs. normal). ANOVA (for continuous variables) and Pearson's chi‐squared test (for categorical variables) were used to determine differences across the three groups.

Adjusted multinomial logistic regression models were fit to determine associations between demographic information, clinical symptoms and exam, EDX testing, neuropathy severity, fiber type involvement, and Camdessanche positivity (sensory length independence) with RFC1 status (biallelic vs. normal, and monoallelic vs. normal).

Specifically, we fit three separate adjusted multinomial logistic regression models to determine the factors associated with RFC1 status. The models included: (1) abnormal findings on clinical exam of the upper and lower extremities and motor and sensory EDX; (2) abnormal findings on clinical exam of upper extremities only and motor and sensory EDX; and (3) polyneuropathy pattern and sensory neuronopathy status. All models were adjusted for age at enrollment in the registry, sex, neuropathy duration, presence of pain, weakness, imbalance, and TNSr score. For each model, pairwise and overall area under the receiver operating characteristic curve (AUC) were calculated to assess the discriminatory capability of the models.

Statistical significance for all tests was determined using two‐sided *p*‐values with a threshold of < 0.05 and all analyses were completed using R version 4.5.0. Missing data was handled with a complete case analysis with respect to each outcome, separately.

### 2.5 Standard Protocol Approvals, Registrations, and Participation Consents

PNRR is approved by each site's Institutional Review Boards. All participants provided written informed consent prior to enrollment.

## 3 Results

### 3.1 Participation and Genetic Testing Information

**FIGURE 1:**
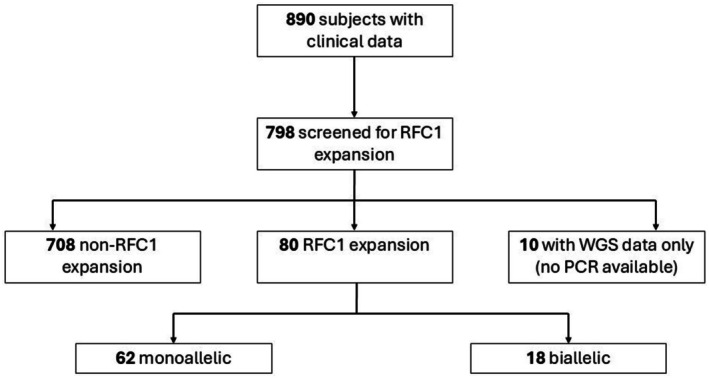
Participant flow chart.

A total of 788 participants were enrolled in the study, of whom 18 had biallelic RFC1 expansions (2.3%), 62 had monoallelic RFC1 expansions (7.9%), and 708 had no expansion (89.8%) (Figure 1). With regards to RFC1 biallelic prevalence across EDX neuropathy phenotypes, it was found that 7.5% (8/106) of pure sensory, 2.3% (7/298) of sensorimotor, and 0% (0/47) of pure motor neuropathy participants demonstrated biallelic expansion. RFC1 monoallelic expansion was seen in 4.7% (5/106) of pure sensory, 6.7% (20/298) of sensorimotor, and 19.1% (9/47) of pure motor neuropathy participants.

### 3.2 Demographic Information and Missing Data

The mean (standard deviation [SD]) age was 62.2 (13.8) years, 61.7% were male, and 93.5% were White. Additional participant characteristics are summarized in Table 1 (see Supplemental Table 1 for additional baseline characteristics). Missing data were notable for the following: glycemic status (*n* = 32), metabolic syndrome (*n* = 102), allodynia (*n* = 233), self‐reported gait imbalance (*n* = 7), motor exam weakness (*n* = 10), Romberg (*n* = 5), sensory EDX abnormality (*n* = 39), motor EDX abnormality (*n* = 36), and EDX polyneuropathy pattern (*n* = 51).

**TABLE 1:** Demographics, metabolic profile, and neuropathy phenotype.

	Overall	Biallelic	Monoallelic	Normal	*p*
	*N* = 788	*N* = 18	*N* = 62	*N* = 708	
Age, mean (SD)	62.2 (13.8)	65.4 (9.6)	62.3 (14.0)	62.1 (13.8)	0.80
Sex, *n* (%)					
Male	484 (61.7%)	9 (50.0%)	35 (58.3%)	440 (62.3%)	0.49
Female	300 (38.3%)	9 (50.0%)	25 (41.7%)	266 (37.7%)	
Race, *n* (%)					
American Indian/Alaska Native	1 (0.1%)	0 (0.0%)	0 (0.0%)	1 (0.1%)	0.64
Asian	10 (1.3%)	0 (0.0%)	0 (0.0%)	10 (1.4%)	
Black or African American	33 (4.2%)	0 (0.0%)	0 (0.0%)	33 (4.7%)	
White	732 (93.5%)	18 (100.0%)	60 (100.0%)	654 (92.8%)	
Metabolic profile					
BMI, mean (SD)	28.2 (5.7)	25.2 (4.6)	27.2 (5.2)	28.3 (5.8)	**0.02**
HbA1C, mean (SD)	5.5 (0.5)	5.7 (0.5)	5.4 (0.4)	5.5 (0.5)	0.38
MetS (*Y*/*N*), *n* (%)	232 (33.8%)	3 (18.8%)	21 (36.8%)	208 (33.9%)	0.39
PN patterns, *n* (%), EDX					
Motor	47 (6.4%)	0 (0.0%)	9 (15.8%)	38 (5.7%)	**< 0.001**
Normal	286 (38.8%)	3 (16.7%)	23 (40.4%)	260 (39.3%)	
Sensorimotor	298 (40.4%)	7 (38.9%)	20 (35.1%)	271 (40.9%)	
Sensory	106 (14.4%)	8 (44.4%)	5 (8.8%)	93 (14.0%)	
PN duration, years (mean [SD])	6.5 (6.4)	7.4 (6.2)	4.8 (4.7)	6.6 (6.6)	0.10

*Note:* Bold values indicate statistical significance based on a two‐sided *p*‐value (< 0.05) from ANOVA/Pearson's chi‐squared test stratified by RFC1 status.

Abbreviations: BMI, body mass index; EDX, electrodiagnostic testing; MetS, metabolic syndrome; PN, peripheral neuropathy.

### 3.3 Comparison of Bi‐ and Mono‐Allelic RFC1 Expansion Participants to Those Without Expansions

The average time since neuropathy symptom onset did not differ significantly between RFC1 biallelic participants (7.4 years [SD 6.2]), RFC1 monoallelic participants (4.8 years [SD 4.7]), and those without expansions (6.6 years [SD 6.6]) (*p* = 0.10). There were also no significant differences in age, sex, race, ethnicity, or metabolic syndrome status between the three cohorts, with the exception of body mass index (BMI), which was higher in non‐biallelic participants (25.2 [4.6] [biallelic] vs. 27.2 [5.2] [monoallelic] vs. 28.3 [5.8] [without expansions], *p* = 0.02). Biallelic participants were more likely to have a mixed fiber polyneuropathy phenotype on physical examination compared to monoallelic participants or those without expansions (88.9% vs. 38.7% vs. 55.6%, *p* = 0.01).

**FIGURE 2:**
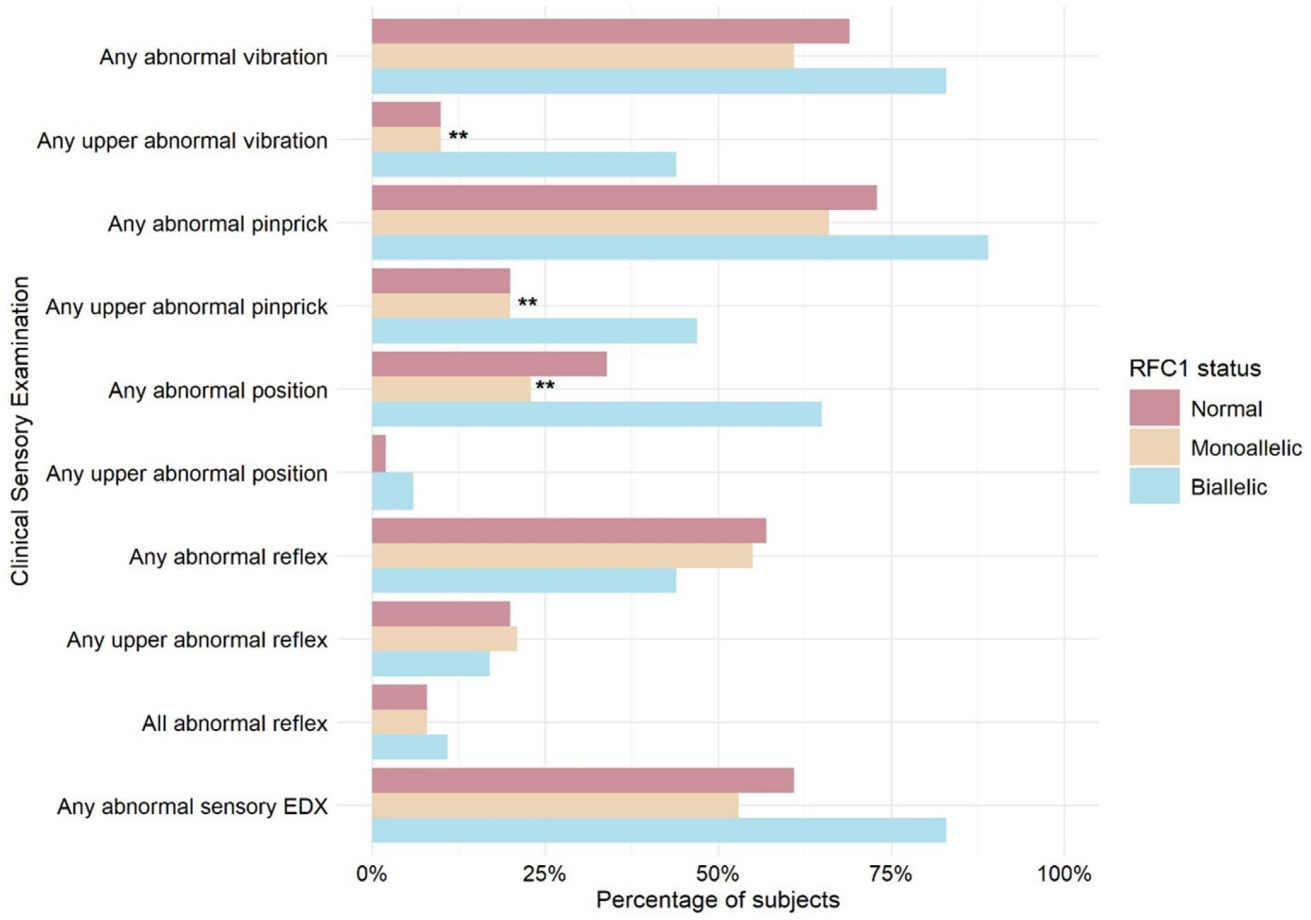
Clinical sensory neurological exam stratified by RFC1 status. **P < 0.05.

**FIGURE 3:**
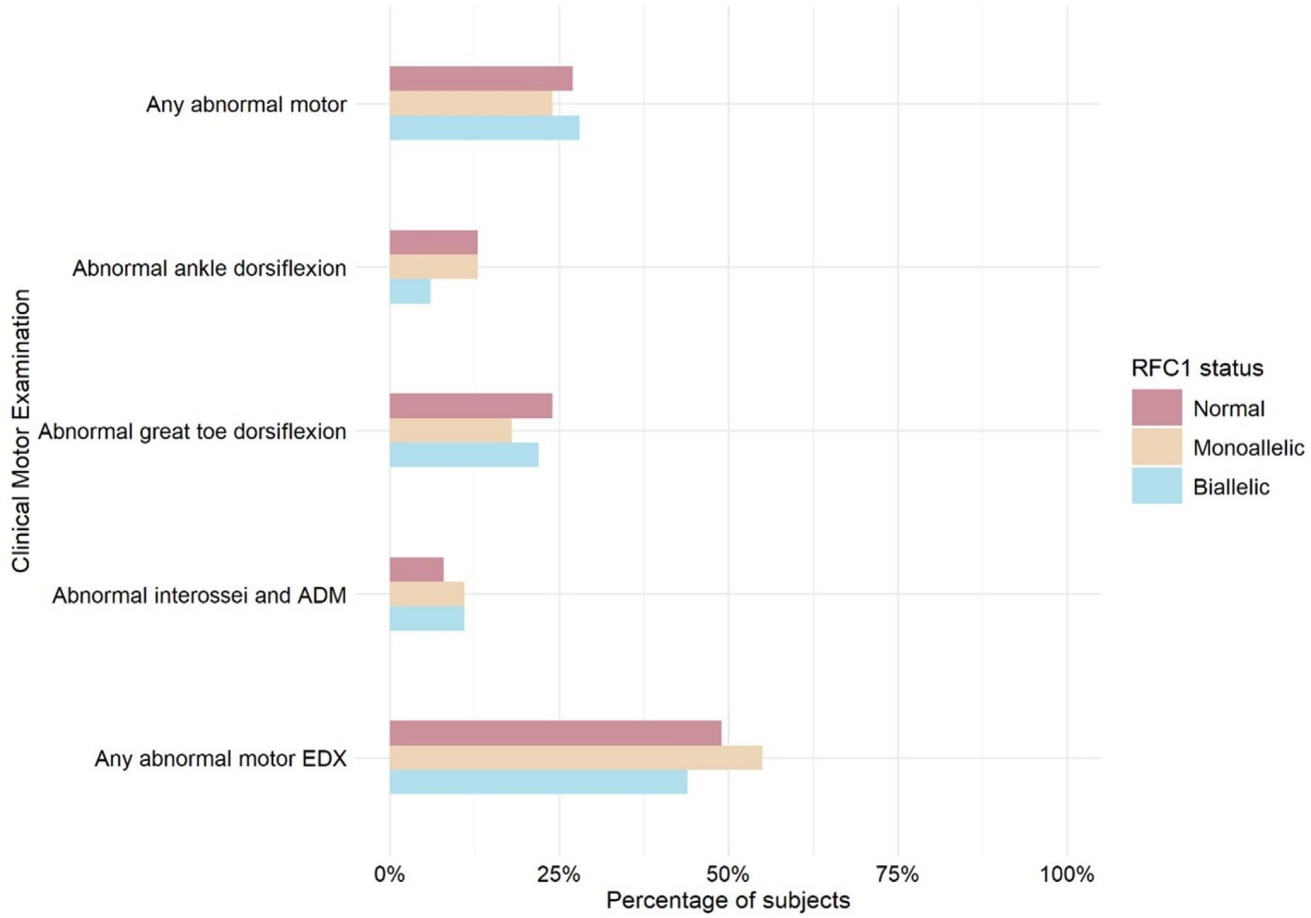
Clinical motor neurological exam stratified by RFC1 status.

**FIGURE 4:**
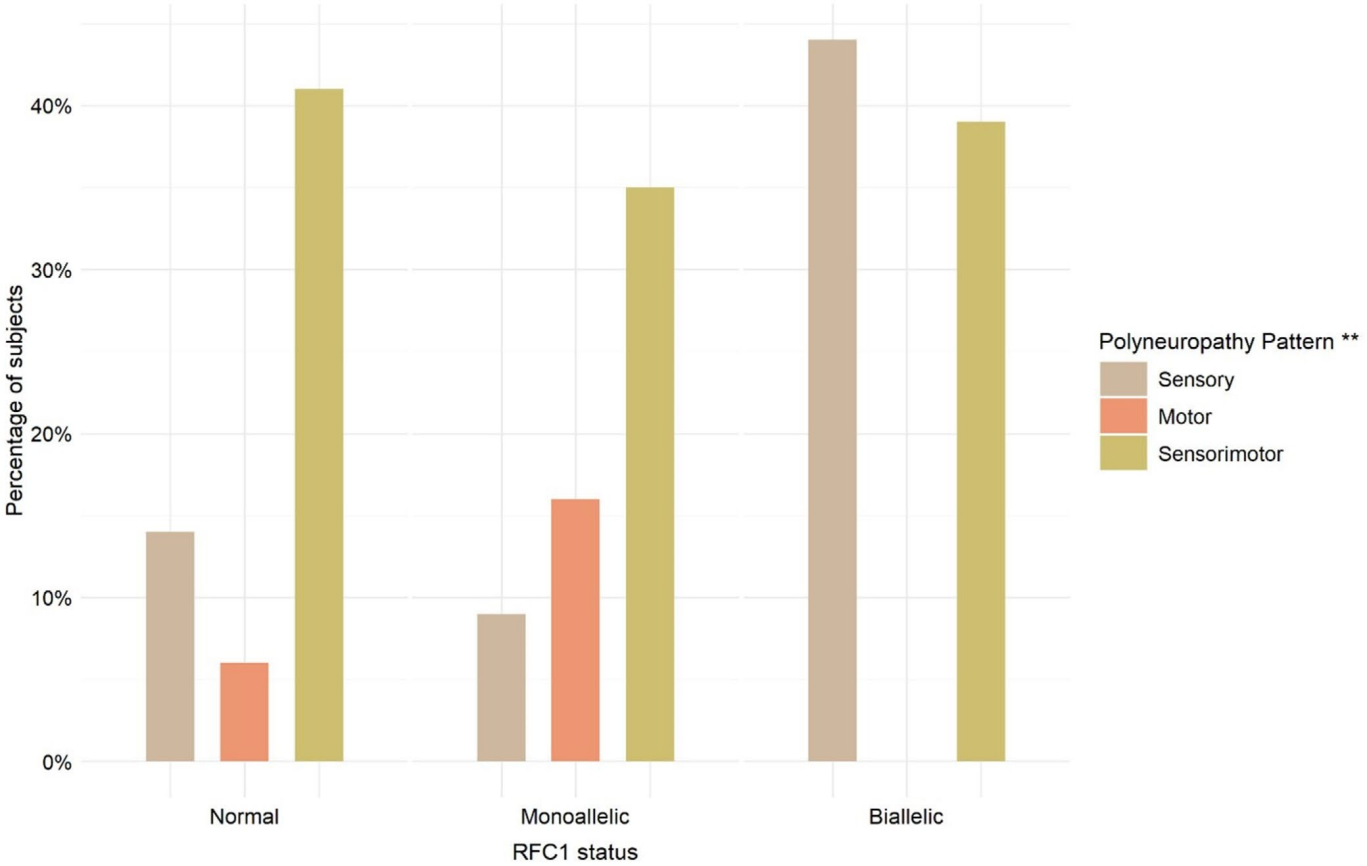
Distribution of clinical presentation of polyneuropathy patterns stratified by RFC1 status.

On physical examination, biallelic participants were more likely, as compared to monoallelic participants and those without expansions, to have abnormal position sensation (upper or lower limb) (64.7% vs. 22.6% vs. 33.9%, *p* = 0.01) as well as abnormal upper limb vibration (44.4% vs. 9.7% vs. 10.2%, *p* < 0.01) and upper limb pinprick sensation (47.1% vs. 19.7% vs. 19.8%, *p* = 0.02) (Figure 2). In addition, biallelic participants more often exhibited abnormal Romberg testing (44.4% vs. 17.7% vs. 18.1%, *p* = 0.02). No differences were seen between biallelic participants, monoallelic participants, and those without expansions on strength testing of toe extension (22.2% vs. 17.7% vs. 24.0%, *p* = 0.53), ankle dorsiflexion (5.6% vs. 12.9% vs. 12.6%, *p* = 0.67), or finger abduction (11.1% vs. 11.3% vs. 8.1%, *p* = 0.62), although motor deficits occurred not infrequently in the biallelic group (27.8%) (Figure 3). With regards to EDX testing, biallelic participants were more likely to have a sensory polyneuropathy diagnosed on EDX testing (Figure 4, 44.4% vs. 8.8% vs. 14.0%, *p* < 0.01). Motor EDX deficits were quite common in biallelic participants (44.4%), monoallelic participants (55.2%), and those without expansions (48.7%), although no group differences were seen (*p* = 0.59). As for fulfillment of the Camdessanche criteria for sensory neuronopathy, this occurred more frequently in biallelic participants as compared to monoallelic participants and those without expansions (44.4% vs. 8.1% vs. 13.1%, *p* < 0.01). As for patterns of sensory nerve action potential loss on EDX (in patients with pure sensory and sensorimotor polyneuropathy), non‐length dependent sensory nerve action potential loss was more frequent in the biallelic group as compared to the monoallelic group or those without expansions (26.7% vs. 0.0% vs. 3.6%, *p* < 0.01).

### 3.4 Multivariable Analysis

Multivariable analyses were conducted to evaluate phenotypic factors associated with RFC1 biallelic and monoallelic status (Tables 2, 3, 4).

**TABLE 2:** Adjusted multinomial logistic regression models (clinical examination in upper/lower extremity, sensory and motor EDX).

Variables	Normal vs. Biallelic	Normal vs. Monoallelic	
	OR (LCI, UCI)	OR (LCI, UCI)	
	AUC = 0.80	AUC = 0.71	Overall AUC = 0.75
Age at enrollment	1.00 (0.96, 1.04)	1.00 (0.98, 1.03)	
Sex—Female	1.41 (0.46, 4.35)	1.17 (0.63, 2.20)	
BMI	0.88 (0.78, 0.98)*	0.97 (0.92, 1.02)	
PN duration	0.97 (0.89, 1.06)	0.93 (0.86, 0.99)*	
TNSr	0.96 (0.83, 1.12)	1.05 (0.96, 1.15)	
Pain	1.62 (0.49, 5.36)	0.83 (0.44, 1.56)	
Weakness	0.58 (0.18, 1.89)	0.52 (0.26, 1.01)	
Imbalance	2.77 (0.58, 13.26)	2.46 (1.19, 5.06)*	
Any abnormality in walk	2.10 (0.58, 7.58)	0.74 (0.34, 1.61)	
Any abnormal pinprick	2.32 (0.45, 11.96)	0.93 (0.48, 1.80)	
Any abnormal vibration	0.92 (0.19, 4.53)	0.72 (0.34, 1.52)	
Any abnormal position	3.11 (0.82, 11.81)	0.6 (0.26, 1.40)	
All abnormal reflex	1.21 (0.22, 6.48)	1.58 (0.54, 4.59)	
Any abnormal motor	0.55 (0.13, 2.34)	0.79 (0.34, 1.86)	
Any abnormal motor EDX	0.39 (0.10, 1.5)	2.45 (1.13, 5.31)*	
Any abnormal sensory EDX	4.44 (0.97, 20.34)	0.48 (0.21, 1.11)	

*Note:* *P < 0.05.

Abbreviations: BMI, body mass index; EDX, electrodiagnostic testing; PN, peripheral neuropathy; TNSr, Total Neuropathy Score, reduced.

**TABLE 3:** Adjusted multinomial logistic regression models (clinical examination in upper extremity, sensory and motor EDX).

Variables	Normal vs. Biallelic	Normal vs. Monoallelic	
	OR (LCI, UCI)	OR (LCI, UCI)	
	AUC = 0.81	AUC = 0.71	Overall AUC = 0.75
Age at enrollment	1.01 (0.96, 1.06)	1.00 (0.98, 1.02)	
Sex—Female	1.57 (0.51, 4.84)	1.13 (0.60, 2.12)	
BMI	0.90 (0.8, 1.01)	0.96 (0.91, 1.02)	
PN duration	0.97 (0.88, 1.06)	0.92 (0.86, 0.99)	
TNSr	0.95 (0.82, 1.11)	1.04 (0.95, 1.13)	
Pain	1.25 (0.37, 4.22)	0.91 (0.48, 1.74)	
Weakness	0.45 (0.13, 1.55)	0.51 (0.26, 0.99)*	
Imbalance	3.28 (0.70, 15.44)	2.35 (1.14, 4.83)*	
Any abnormality in walk	1.70 (0.47, 6.13)	0.61 (0.28, 1.31)	
Any upper abnormal pinprick	2.87 (0.93, 8.86)	1.16 (0.53, 2.56)	
Any upper abnormal vibration	4.57 (1.20, 17.36)*	1.15 (0.39, 3.35)	
Any upper abnormal position	1.38 (0.10, 19.26)	—	
All abnormal reflex	1.03 (0.15, 7.13)	1.73 (0.60, 4.99)	
Any abnormal motor	0.67 (0.15, 2.91)	0.72 (0.31, 1.71)	
Any abnormal motor EDX	0.47 (0.13, 1.76)	2.31 (1.07, 5.00)*	
Any abnormal sensory EDX	4.61 (1.04, 20.52)*	0.44 (0.19, 1.02)	

*Note:* *P < 0.05.

Abbreviations: BMI, body mass index; EDX, electrodiagnostic testing; PN, peripheral neuropathy; TNSr, Total Neuropathy Score, reduced.

**TABLE 4:** Adjusted multinomial logistic regression models (polyneuropathy pattern and Camdessanche positive).

Variables	Normal vs. Biallelic	Normal vs. Monoallelic	
	OR (LCI, UCI)	OR (LCI, UCI)	
	AUC = 0.81	AUC = 0.69	Overall AUC = 0.75
Age at enrollment	1.00 (0.95, 1.04)	1.00 (0.98, 1.02)	
Sex—Female	1.68 (0.58, 4.85)	1.17 (0.64, 2.16)	
BMI	0.88 (0.79, 0.98)*	0.96 (0.91, 1.02)	
PN duration	0.99 (0.92, 1.08)	0.92 (0.86, 0.99)*	
TNSr	0.99 (0.87, 1.12)	1.04 (0.96, 1.12)	
Pain	1.58 (0.48, 5.18)	0.86 (0.46, 1.61)	
Weakness	0.58 (0.19, 1.8)	0.53 (0.27, 1.01)	
Imbalance	2.82 (0.65, 12.29)	2.31 (1.14, 4.70)*	
Polyneuropathy pattern (Ref: normal)			
Motor	—	2.62 (1.04, 6.57)*	
Sensorimotor	3.25 (0.52, 20.29)	0.79 (0.31, 2.06)	
Sensory	7.73 (1.68, 35.51)*	0.71 (0.25, 2.06)	
Camdessanche positive—Yes	3.53 (1.15, 10.84)*	0.48 (0.16, 1.43)	

*Note:* *P < 0.05.

Abbreviations: BMI, body mass index; PN, peripheral neuropathy; TNSr, Total Neuropathy Score, reduced.

Multinomial regression models (model 1) revealed that higher BMI was associated with lower odds of RFC1 biallelic status as compared to non‐expansion (OR 0.88, 95% CI 0.78–0.98). With regards to physical examination (model 2), abnormal upper limb vibration (biallelic expansion vs. non‐expansion: OR 4.57, 95% CI 1.20–17.36) was the only variable associated with RFC1 biallelic expansion. In model 3, fulfillment of the Camdessanche sensory neuronopathy criteria was associated with biallelic status (OR 3.53, 95% CI 1.15–10.84). With regards to EDX (model 2), the presence of sensory EDX abnormality (OR 4.61, 95% CI 1.04–20.52) and the presence of a pure sensory polyneuropathy on EDX (OR 7.73, 95% CI 1.68–35.51) both were associated with biallelic status. With AUCs ranging from 0.80 to 0.81, the models had “good” ability to discriminate biallelic status from those without expansion.

With regards to RFC1 monoallelic status, neuropathy duration (monoallelic expansion vs. non‐expansion: OR 0.93, 95% CI 0.86–0.99), self‐reported weakness (OR 0.51, 95% CI 0.26–0.99), and self‐reported gait imbalance (OR 2.46, 95% CI 1.19–5.06) were associated. On EDX, the presence of any abnormal EDX motor parameter (OR 2.45, 95% CI 1.13–5.31) and the presence of a pure motor neuropathy (OR 2.62, 95% CI 1.04–6.57) both also associated. In contrast to biallelic participants, the models for monoallelic participants had “poor” to “fair” ability to discriminate monoallelic expansion status from those without expansion.

## 4 Discussion

Compared with the two previously reported 2021 European cohorts, our North American cohort demonstrated a markedly lower prevalence of biallelic RFC1 expansion and was slightly lower than prevalence data from New Zealand. In addition, motor involvement, as defined by EDX testing, was more prevalent than previously seen in any cohort. Nevertheless, our ability to discriminate biallelic status compared to no expansion is good, suggesting that targeted genetic testing might be possible.

RFC1 biallelic prevalence in our total CIAP population was only 2.3%, with a 7.5% prevalence amongst pure sensory neuropathy and 2.3% amongst sensorimotor participants. Pure sensory neuropathy prevalence was markedly lower than that reported in Curro (34%) and Tagliapietra (53%), but more comparable to the New Zealand cohorts, which screened only pure sensory and sensory predominant CIAP participants, and showed a prevalence rate of 10.3%–11.6% [8, 9]. With regards to sensorimotor neuropathy, the Curro study showed a prevalence of 0% (0/100). The Tagliapietra study showed 2% prevalence in sensorimotor and 18% prevalence in predominantly sensory neuropathy, also closer to our prevalence rate. Some of the discrepancy could be traced to how neuropathy is defined, with the Curro study employing an EDX based definition (like ours) and the Tagliapietra study using a physical exam‐based phenotype. In the Tagliapietra study, sensorimotor neuropathy was defined as Medical Research Council score ≤ 3/5 on bilateral muscle strength testing and sensory predominant neuropathy as bilateral motor weakness not fulfilling that cutoff. In addition to the differing definitions of neuropathy, the inclusion of metabolic syndrome, obesity, diabetes, and prediabetes in our cohort of CIAP may have impacted biallelic prevalence figures, although it should be noted that obesity, metabolic syndrome, and prediabetes were not explicitly excluded in the Curro study nor the Tagliapietra study (obesity, metabolic syndrome, and BMI baseline profiles for the RFC1‐positive and RFC‐negative were not provided). In addition, excluding any participant with obesity, metabolic syndrome, or prediabetes from our CIAP cohort would have led to the removal of a significant number of RFC1 biallelic expansion associated neuropathy participants. Of the 18 total RFC1 biallelic expansion participants in our cohort, 3 had prediabetes, 2 had diabetes, and 3 had metabolic syndrome. Furthermore, subgroup comparison between RFC1 biallelic participants with or without dysmetabolism showed no differences in non‐neuronopathic features (motor involvement, Camdessanche negativity, nor length dependent EDX pattern), suggesting that RFC1 biallelic expansion was likely the main driver of neuropathy in both groups. In addition, while the 2026 Pelosi study from New Zealand highlighted the role of obesity/metabolic syndrome in CIAP as a distinct entity from RFC1 associated neuropathy vis a vis ultrasound [9], the 2025 Garvey study also highlighted that the mere presence of diabetes should not preclude the search for RFC1 expansion [8]. A likely greater concern when comparing prevalence figures for RFC1 neuropathy are population‐level differences in the prevalence of obesity between different study cohorts (USA—41.64%, New Zealand—32.99%, UK—26.94%, Italy—17.97%) [22]. Finally, geographic origin likely impacts the prevalence of RFC1 expansion associated neuropathy, as evidenced by the lower prevalence figures seen in the New Zealand and North American (our) cohorts relative to the continental European cohorts. In short, we are finding a much lower prevalence of biallelic repeats in those with pure sensory neuropathy than previously reported, which has implications for genetic testing within these populations.

Although RFC1 biallelic associated neuropathy is disproportionately sensory in its presentation, motor involvement may not be as rare as previously reported. In our cohort, 61.1% of participants with biallelic RFC1 expansion reported subjective weakness, 27.8% had objective distal weakness on physical examination, 44.4% had abnormalities on motor nerve conduction studies, and 38.9% had a sensorimotor polyneuropathy on EDX testing, all indicating greater motor involvement than previously reported. Therefore, reserving testing for those with sensory neuropathy is likely to miss a significant portion of RFC1 cases. Previous studies had indicated that patients with motor involvement were very unlikely to have biallelic RFC1, with the Curro study finding weakness in 0/43 participants and motor NCS being normal in all but two participants. While data remain mixed, the presence of motor involvement should not preclude consideration for RFC1 biallelic expansion testing in the proper clinical context.

Our multivariable models had good discriminatory ability to detect RFC1 biallelic status (AUCs from the multivariable models ranged from 0.80 to 0.81) based on the presence of abnormal upper limb vibration sensation, the presence of EDX sensory loss, the presence of a sensory polyneuropathy on EDX, fulfillment of Camdessanche criteria, and BMI. The preferential upper limb vibration (and possible pinprick) sensory involvement seen in our study aligns with the Curro 2021 study, which found disproportionate vibratory (81% of participants) and pinprick (65% of participants) sensation loss with relative sparing of position sensation (only 23% of participants). The Tagliapietra study found no difference in tested sensory modalities (pinprick, vibration, or joint position sensation) between RFC1 and non‐RFC1 participants. In agreement with the 2021 Curro trial, our study also supports a role for sensory neuronopathy, as defined by the Camdessanche criteria, as a predictor of biallelic RFC1 expansion status. In our study, 44.4% of our biallelic cohort fulfilled Camdessanche criteria for sensory neuronopathy. In the Curro paper, 44% of biallelic participants had a non‐length dependent reduction in amplitudes on EDX testing, whilst 30% had length dependent sensory loss. The 2025 Garvey study from New Zealand found 4 of the 5 RFC1 biallelic expansion participants (80%) to fulfill possible Camdessanche criteria for sensory neuronopathy, with 4 of 5 (80%) also fulfilling a non‐length dependent pattern of sensory loss on EDX [8]. In our study, 44.4% of participants fulfilled Camdessanche criteria for sensory neuronopathy and 26.7% showed non‐length dependent sensory nerve action potential loss on EDX. In addition, none of the 5 RFC1 biallelic expansion participants in the 2025 Garvey cohort showed motor involvement on EDX, which was defined as “any abnormal upper limb motor amplitude or a reduction of > 50% in a lower limb motor amplitude.” Reasons for such discrepancies between the Garvey and Curro studies and ours remain uncertain but may be due to differences in population demographics, extent of EDX testing and/or sample size. The presence of metabolic syndrome may have also affected length dependent versus non‐length dependent classification, although uncertain. Difference in prevalence of motor EDX involvement between our study and the 2025 Garvey study is very likely due to differing criteria to define motor involvement (we did not require a 50% amplitude reduction cutoff in lower limb motor responses). We did not find, however, an increased likelihood of gait ataxia (Romberg, gait) in our cohort of biallelic participants, although Romberg testing abnormality was more common in biallelic participants as compared to those without expansions on univariate analysis. BMI, the fifth and final multivariable predictor of RFC1 biallelic expansion status in our study, was found to be higher in non‐biallelic participants (i.e., less altered in biallelic participants). This appears to align with the 2026 Pelosi study from New Zealand that found RFC1 biallelic participants to more frequently have normal/mildly altered BMI as compared to RFC1‐negative CIAP participants [9]. The reason for such a potential association between BMI and RFC1 biallelic expansion neuropathy remains unclear, but worthy of further investigation. Finally, it has been shown that in certain subpopulations of CIAP, such as hereditary sensory and autonomic neuropathy (HSAN) (who are whole exome sequence negative), the concomitant presence of chronic, inexplicable cough should prompt testing for RFC1, as prevalence rates may be as high as 75% [23].

Finally, our study uniquely assessed factors associated with monoallelic RFC1 (or carrier) status. We found neuropathy duration, self‐reported weakness, self‐reported gait imbalance, and motor abnormalities on EDX (including the presence of a pure motor neuropathy on EDX) to be associated with monoallelic status, albeit not in a consistent direction. When comparing the baseline characteristics of monoallelic participants and those without expansions, clinical features were quite similar. Furthermore, the frequency of RFC1 monoallelic expansion in our CIAP cohort (8%) is comparable to the allele frequency in large population databases (4.5%) [24]. In addition, models struggled to discriminate participants with monoallelic RFC1 expansion versus those without expansion. While the role of monoallelic expansion status in neuropathy is worthy of future investigation, our study seems to argue against its role as a cause of neuropathy.

Given the retrospective nature of the registry, our study could not capture other dimensions associated with RFC1 repeat expansion disorders and CANVAS, namely appendicular ataxia, cerebellar ataxia, vestibular areflexia, and autonomic dysfunction. In particular, ultrasonographic nerve cross‐sectional area, a promising new biomarker and predictor of RFC1 expansion status, as well as the presence/absence of chronic cough, were not available/included for our cohort. Importantly, our CIAP cohort allowed for the inclusion of metabolic syndrome, obesity, and prediabetes, which are known drivers of neuropathy, and thus may have inadvertently led to falsely low RFC1 prevalence rates and/or impacted neuropathy phenotype. However, it remains unclear as to whether such variables (particularly metabolic syndrome and obesity), when present, should preclude testing for RFC1 expansion (on the basis that the neuropathy is not truly idiopathic). Despite its strength, our predictive model would still likely miss a subset of RFC1 cases. Also, our finding of increased motor involvement in RFC1 expansion associated neuropathy is tempered by the fact that we did not grade severity of muscle weakness on strength testing (only its presence/absence). In addition, the Camdessanche criteria employed in this study to define sensory neuronopathy may fall short of accurate identification of RFC1 biallelic cases, as they the original criteria were derived from paraneoplastic sensory neuronopathies and not sensory neuronopathies in general. From a demographic standpoint, our cohort was all recruited from one US academic center and were largely White; therefore, generalizability to other populations is unclear. Our sample size also limited our power to detect small associations; however, we were able to determine multiple significant associated variables for both biallelic and monoallelic RFC1 status.

In conclusion, biallelic RFC1 expansion status appears less prevalent than previously reported. In addition, motor fiber involvement may be higher than previously thought. Thus, testing should not be limited to participants with sensory only neuropathy. However, models including the presence of a clinical phenotype characterized by preferential upper limb vibratory loss on exam, disproportionate sensory EDX injury, sensory only polyneuropathy on EDX, fulfillment of the Camdessanche sensory neuronopathy criteria, and BMI can discriminate between RFC1 biallelic status and non‐carriers; therefore, it may be possible to determine who should warrant dedicated genetic testing. At present, evidence is lacking for a pathogenic role for RFC1 monoallelic expansion as a cause of neuropathy, which has a similar phenotype to non‐carriers.

## Author Contributions

**Amro M. Stino:** conceptualization, methodology, investigation, writing – original draft, visualization, project administration. **Lavanya Muthukumar:** methodology, software, formal analysis, writing – original draft. **Evan L. Reynolds:** methodology, software, formal analysis, writing – review and editing. **Peter Todd:** conceptualization, methodology, investigation, writing – review and editing. **Sinem Ovunc:** methodology, investigation. **Sheng Chih Jin:** conceptualization, methodology, investigation, writing – review and editing. **Zitian Tang:** conceptualization, methodology, investigation, writing – review and editing. **Simone Thomas:** conceptualization, investigation, resources, writing – review and editing, project administration. **Ahmet Höke:** conceptualization, methodology, investigation, writing – review and editing. **Brian C. Callaghan:** conceptualization, methodology, formal analysis, writing – review and editing, visualization, supervision.

## Funding

E.L.R. is supported by the NIH NIDDK (R00DK129785). PNRR Study Group is supported by the Foundation for Peripheral Neuropathy. A.H. is supported by NIH (R21NS135481, P30 MH075673‐011), Wellcome Trust, Dr. Miriam and Sheldon G. Adelson Medical Research Foundation, and Dr. Richard Merkin Family Foundation. S.C.J. is supported by NIH U19NS130607.

## Conflicts of Interest

A.M.S. has consulted for Argenx, CSL Behring, Takeda, Sanofi, Immunovant, Annexon, and has received research support from Bristol Myers Squibb and the GBS‐CIDP Foundation. A.H. consults for Pfizer, Sanofi, Novartis, Roche, Sangamo, GenEdit, Eikonizo, HDAX Therapeutics and Sangamo and receives editorial support from the American Neurological Association and research funding from Sanofi, DOD, NINDS, Dr. Miriam and Sheldon G. Adelson Medical Research Foundation, Merkin Family Foundation and Foundation for Peripheral Neuropathy. B.C.C. consults for DynaMed, performs medical legal consultations, including consultations for the Vaccine Injury Compensation Program, and receives research and editorial support from the American Academy of Neurology. L.M., E.L.R., P.T., S.O., S.C.J., and Z.T. report no conflicts of interest to disclose.

## Supporting information


**Supplemental Table 1.** Demographics, metabolic profile, and neuropathy clinical and electrodiagnostic characteristics.

## Data Availability

The data that support the findings of this study are available from the Peripheral Neuropathy Research Registry (PNRR). Restrictions apply to the availability of these data, which were used under license for this study. Data are available from the authors at the URL with the permission of the PNRR and the Foundation for Peripheral Neuropathy.
