# Supplementary material for: Bi‐ and Mono‐Allelic RFC1 Expansion in a North American Cohort With Idiopathic Axonal Neuropathy
Source: Ann Clin Transl Neurol. 2026 Jul 5:10.1002/acn3.70462. Online ahead of print. doi: 10.1002/acn3.70462 (PMC13394504; doi:10.1002/acn3.70462)
Supplement: Supplementary file 1 — Supplemental Table 1. Demographics, metabolic profile, and neuropathy clinical and electrodiagnostic characteristics. [file ACN3-9999-0-s001.docx]

**Supplemental Table 1: Demographics, metabolic profile, and neuropathy clinical and electrodiagnostic characteristics**

|  | **Missing N** | **Overall** | **Biallelic** | **Monoallelic** | **Normal** | **p-value** |
| --- | --- | --- | --- | --- | --- | --- |
|  |  | **N = 788** | **N = 18** | **N = 62** | **N = 708** |  |
| **Age,** mean (sd) | - | 62.2 (13.8) | 65.4 (9.6) | 62.3 (14.0) | 62.1 (13.8) | 0.80 |
| **Sex n(%)** | 4 |  |  |  |  | 0.49 |
| Male |  | 484 (61.7%) | 9 (50.0%) | 35 (58.3%) | 440 (62.3%) |  |
| Female |  | 300 (38.3%) | 9 (50.0%) | 25 (41.7%) | 266 (37.7%) |  |
| **Race n(%)** | 5 |  |  |  |  | 0.64 |
| American Indian/ Alaska Native |  | 1 (0.1%) | 0 (0.0%) | 0 (0.0%) | 1 (0.1%) |  |
| Asian |  | 10 (1.3%) | 0 (0.0%) | 0 (0.0%) | 10 (1.4%) |  |
| Black or African American |  | 33 (4.2%) | 0 (0.0%) | 0 (0.0%) | 33 (4.7%) |  |
| White |  | 732 (93.5%) | 18 (100.0%) | 60 (100.0%) | 654 (92.8%) |  |
| More than one race |  | 7 (0.9%) | 0 (0.0%) | 0 (0.0%) | 7 (1.0%) |  |
| **Hispanic n(%)** - Yes | 6 | 17 (2.2%) | 1 (5.6%) | 0 (0.0%) | 16 (2.3%) | 0.31 |
| **METABOLIC FACTORS** |  |  |  |  |  |  |
| Systolic BP, mean (sd) | 37 | 129.8 (17.2) | 127.7 (16.0) | 130.7 (17.9) | 129.7 (17.2) | 0.91 |
| Diastolic BP, mean (sd) | 37 | 75.4 (10.1) | 73.8 (9.7) | 75.2 (10.1) | 75.5 (10.1) | 0.83 |
| Triglycerides, mean (sd) | 278 | 124.0 (97.9) | 102.4 (68.8) | 114.8 (62.1) | 125.4 (101.2) | 0.44 |
| HDL, mean (sd) | 281 | 56.1 (18.9) | 62.8 (18.7) | 56.5 (16.7) | 55.9 (19.1) | 0.35 |
| BMI, mean (sd) | 1 | 28.2 (5.7) | 25.2 (4.6) | 27.2 (5.2) | 28.3 (5.8) | **0.02** |
| HbA1C, mean (sd) | 155 | 5.5 (0.5) | 5.7 (0.5) | 5.4 (0.4) | 5.5 (0.5) | 0.38 |
| **Glycemic status n(%)** | 32 |  |  |  |  |  |
| Normoglycemia |  | 525 (69.4%) | 10 (66.7%) | 43 (70.5%) | 472 (69.4%) | 0.53 |
| Prediabetes |  | 198 (26.2%) | 3 (20.0%) | 16 (26.2%) | 179 (26.3%) |  |
| Diabetes |  | 33 (4.4%) | 2 (13.3%) | 2 (3.3%) | 29 (4.3%) |  |
| **Mets** n(%) (mets >= 3) | 102 |  |  |  |  |  |
| Yes |  | 232 (33.8%) | 3 (18.8%) | 21 (36.8%) | 208 (33.9%) | 0.39 |
| **NEUROPATHY PROFILE** |  |  |  |  |  |  |
| Fiber type n(%) | - |  |  |  |  |  |
| Large |  | 106 (13.5%) | 0 (0.0%) | 12 (19.4%) | 94 (13.3%) | **0.01** |
| Mixed |  | 434 (55.1%) | 16 (88.9%) | 24 (38.7%) | 394 (55.6%) |  |
| Small |  | 123 (15.6%) | 0 (0.0%) | 15 (24.2%) | 108 (15.3%) |  |
| Unknown |  | 125 (15.9%) | 2 (11.1%) | 11 (17.7%) | 112 (15.8%) |  |
| **Symptom onset and duration** |  |  |  |  |  |  |
| PN duration |  | 6.5 (6.4) | 7.4 (6.2) | 4.8 (4.7) | 6.6 (6.6) | 0.10 |
| Years since onset of PN | 16 | 6.4 (6.4) | 7.4 (6.2) | 4.7 (4.8) | 6.5 (6.6) | 0.09 |
| Years since first noticed PN symptoms | 6 | 6.5 (6.5) | 7.4 (6.2) | 4.8 (4.7) | 6.6 (6.6) | 0.1 |
| **Taking PN Medications** n(%) | 288 |  |  |  |  |  |
| Yes |  | 321 (64.2%) | 5 (55.6%) | 22 (59.5%) | 294 (64.8%) | 0.21 |
| **Total Neuropathy score** (mean (SD)) | - | 8.8 (5.4) | 10.4 (5.4) | 8.4 (5.2) | 8.8 (5.4) | 0.3 |
| **SYMPTOMS** |  |  |  |  |  |  |
| **Pain** n(%) | - |  |  |  |  |  |
| Yes |  | 558 (70.8%) | 14 (77.8%) | 41 (66.1%) | 503 (71.0%) | 0.58 |
| **Pain Intensity** (0-10) (mean (SD)) |  | 5.6 (2.5) | 6.1 (2.2) | 5.5 (2.8) | 5.6 (2.4) | 0.82 |
| **Allodynia n(%)** | 232 |  |  |  |  |  |
| Yes |  | 273 (49.1%) | 7 (50.0%) | 22 (53.7%) | 244 (48.7%) | 0.83 |
| **Numbness** n(%) | - |  |  |  |  |  |
| Yes |  | 692 (87.8%) | 16 (88.9%) | 55 (88.7%) | 621 (87.7%) | 0.96 |
| **Sensation n(%)** | 10 |  |  |  |  |  |
| No |  | 117 (15.0%) | 2 (11.1%) | 7 (11.7%) | 108 (15.4%) | 0.44 |
| Yes all the time |  | 294 (37.8%) | 4 (22.2%) | 22 (36.7%) | 268 (38.3%) |  |
| Yes occasionally |  | 367 (47.2%) | 12 (66.7%) | 31 (51.7%) | 324 (46.3%) |  |
| **Contractions n(%)** | 11 |  |  |  |  |  |
| No |  | 213 (27.4%) | 4 (22.2%) | 22 (36.7%) | 187 (26.8%) | 0.70 |
| Frequently |  | 132 (17.0%) | 4 (22.2%) | 8 (13.3%) | 120 (17.2%) |  |
| Sometimes |  | 254 (32.7%) | 5 (27.8%) | 19 (31.7%) | 230 (32.9%) |  |
| Rarely |  | 178 (22.9%) | 5 (27.8%) | 11 (18.3%) | 162 (23.2%) |  |
| **Imbalance n(%)** | 7 |  |  |  |  |  |
| Yes |  | 479 (61.3%) | 15 (83.3%) | 38 (63.3%) | 426 (60.6%) | 0.14 |
| **Weakness n(%)** | 10 |  |  |  |  |  |
| Yes |  | 421 (54.1%) | 11 (61.1%) | 27 (45.0%) | 383 (54.7%) | 0.29 |
| **Gait n**(%) | 2 |  |  |  |  |  |
| Abnormal |  | 163 (20.7%) | 7 (38.9%) | 9 (14.5%) | 147 (20.8%) | 0.08 |
| **Tandem n(%)** | 9 |  |  |  |  |  |
| Not Able |  | 235 (30.2%) | 9 (50.0%) | 15 (24.2%) | 211 (30.2%) | 0.11 |
| **Romberg n(%)** | 5 |  |  |  |  |  |
| Present/ Positive |  | 146 (18.6%) | 8 (44.4%) | 11 (17.7%) | 127 (18.1%) | **0.02** |
| **SENSORY** |  |  |  |  |  |  |
| **Any pinprick abnormal** n(%) | - |  |  |  |  |  |
| Yes |  | 574 (72.8%) | 16 (88.9%) | 41 (66.1%) | 517 (73.0%) | 0.15 |
| **Any abnormal pinprick upper extremity n(%)** | 2 |  |  |  |  |  |
| Yes |  | 160 (20.4%) | 8 (47.1%) | 12 (19.7%) | 140 (19.8%) | **0.02** |
| **Any vibration abnormal** n(%) | 1 |  |  |  |  |  |
| Yes |  | 539 (68.5%) | 15 (83.3%) | 38 (61.3%) | 486 (68.7%) | 0.19 |
| **Any abnormal vibration upper extremity n(%)** | 2 |  |  |  |  |  |
| Yes |  | 86 (10.9%) | 8 (44.4%) | 6 (9.7%) | 72 (10.2%) | **<0.001** |
| **Any position abnormal** n(%) | 1 |  |  |  |  |  |
| Yes |  | 265 (33.7%) | 11 (64.7%) | 14 (22.6%) | 240 (33.9%) | **0.01** |
| **Any abnormal position upper extremity n(%)** |  |  |  |  |  |  |
| Yes | 1 | 13 (1.7%) | 1 (5.9%) | 0 (0.0%) | 12 (1.7%) | 0.23 |
| Any reflex abnormal n(%) | - |  |  |  |  |  |
| Yes |  | 444 (56.3%) | 8 (44.4%) | 34 (54.8%) | 402 (56.8%) | 0.56 |
| **Any reflex abnormal upper extremity n(%)** | - |  |  |  |  |  |
| Yes |  | 161 (20.4%) | 3 (16.7%) | 13 (21.0%) | 145 (20.5%) | 0.92 |
| **All reflex abnormal** (>=2 abnormal & 0 normal) **n(%)** | - |  |  |  |  |  |
| Yes |  | 62 (7.9%) | 2 (11.1%) | 5 (8.1%) | 55 (7.8%) | 0.87 |
| **Any sensory edx abnormal n(%)** | 39 |  |  |  |  |  |
| Yes |  | 459 (61.3%) | 15 (83.3%) | 30 (52.6%) | 414 (61.4%) | 0.06 |
| **MOTOR** |  |  |  |  |  |  |
| **Any motor edx abnormal n(%)** | 36 |  |  |  |  |  |
| Yes |  | 369 (49.1%) | 8 (44.4%) | 32 (55.2%) | 329 (48.7%) | 0.59 |
| **Any motor abnormal n(%)** | - |  |  |  |  |  |
| Yes |  | 214 (27.2%) | 5 (27.8%) | 15 (24.2%) | 194 (27.4%) | 0.86 |
| **Abnormal ankle dorsiflexion n(%)** | - |  |  |  |  |  |
| Yes |  | 98 (12.4%) | 1 (5.6%) | 8 (12.9%) | 89 (12.6%) | 0.67 |
| **Abnormal motor toe extension n(%)** | - |  |  |  |  |  |
| Yes |  | 185 (23.5%) | 4 (22.2%) | 11 (17.7%) | 170 (24.0%) | 0.53 |
| **Abnormal finger abduction n(%)** | - |  |  |  |  |  |
| Yes |  | 66 (8.4%) | 2 (11.1%) | 7 (11.3%) | 57 (8.1%) | 0.62 |
| **Camdessanche positive n(%) (score > 6.5 )** |  |  |  |  |  |  |
| Yes |  | 106 (13.5%) | 8 (44.4%) | 5 (8.1%) | 93 (13.1%) | **<0.01** |
| **SNAP PATTERN OF LOSS** (only among pure sensory and sensorimotor patterns, N = 404) n(%) |  |  |  |  |  |  |
| Globally absent SNAPs |  | 2 (0.5%) | 1 (6.7%) | 0 (0.0%) | 1 (0.3%) | **<0.01** |
| Length dependent reduction in SNAPs |  | 385 (95.3%) | 10 (66.7%) | 25 (100.0%) | 350 (96.2%) |  |
| Non-length dependent reduction in SNAPs |  | 17 (4.2%) | 4 (26.7%) | 0 (0.0%) | 13 (3.6%) |  |
| **Polyneuropathy patterns n(%)** | 51 |  |  |  |  |  |
| Motor |  | 47 (6.4%) | 0 (0.0%) | 9 (15.8%) | 38 (5.7%) | **<0.001** |
| Normal |  | 286 (38.8%) | 3 (16.7%) | 23 (40.4%) | 260 (39.3%) |  |
| Sensorimotor |  | 298 (40.4%) | 7 (38.9%) | 20 (35.1%) | 271 (40.9%) |  |
| Sensory |  | 106 (14.4%) | 8 (44.4%) | 5 (8.8%) | 93 (14.0%) |  |
